# 

**Published:** 1899-04-08

**Authors:** 


					THE HOSPITAL. "The standard ot highest punty.JJ_LanceL "MWWIffRflF
^OASY's fHi CAD.rs
ABSOLUTELY PURE. NO DRUGS OR ALKALIES USED.
urwich Hospital
No. 65*. Vol. XXVI. [Sa
Saturday, April 8th, 1899.
j^buoscripUon q-L eims, j pRICE TWOPENCE.

				

